# Supplementary material for: MEME-LaB: motif analysis in clusters
Source: Bioinformatics. 2013 May 14;29(13):1696–7. doi: 10.1093/bioinformatics/btt248 (PMC3694638; doi:10.1093/bioinformatics/btt248)
Supplement: Supplementary Data [file supp_btt248_Supplementary_Data.zip › Supplementary_Data/Results_Files/which_gene_ids/cluster12_motif1.html]

Cluster 12 Motif 1


Cluster 12 Motif 1 is found in the following promoters

| Gene ID | Strand | Position\* |
| --- | --- | --- |

|  |  |  |
| --- | --- | --- |
| AT1G01170 | + | 110/500 |
| AT1G01360 | + | 448/500 |
| AT1G02280 | - | 233/500 |
| AT1G04550 | + | 193/500 |
| AT1G08110 | + | 276/500 |
| AT1G08980 | + | 142/500 |
| AT1G09415 | + | 462/500 |
| AT1G09570 | - | 99/500 |
| AT1G10820 | + | 453/500 |
| AT1G11280 | - | 151/500 |
| AT1G12010 | - | 315/500 |
| AT1G13640 | - | 223/500 |
| AT1G15180 | + | 212/500 |
| AT1G16400 | + | 118/500 |
| AT1G17050 | - | 295/500 |
| AT1G17190 | + | 457/500 |
| AT1G17200 | - | 210/500 |
| AT1G18460 | - | 106/500 |
| AT1G18650 | + | 322/500 |
| AT1G19340 | - | 28/500 |
| AT1G19600 | + | 94/500 |
| AT1G22850 | - | 180/500 |
| AT1G23030 | - | 329/500 |
| AT1G23080 | - | 111/500 |
| AT1G24450 | - | 487/500 |
| AT1G28400 | + | 430/500 |
| AT1G29395 | + | 61/500 |
| AT1G29700 | + | 408/500 |
| AT1G29900 | + | 330/500 |
| AT1G31800 | - | 468/500 |
| AT1G34000 | + | 217/500 |
| AT1G45191 | + | 169/500 |
| AT1G45207 | - | 332/500 |
| AT1G47740 | + | 224/500 |
| AT1G48310 | + | 72/500 |
| AT1G49430 | + | 367/500 |
| AT1G49630 | + | 89/500 |
| AT1G49730 | - | 486/500 |
| AT1G49975 | + | 188/500 |
| AT1G50290 | + | 17/500 |
| AT1G51140 | + | 266/500 |
| AT1G52040 | + | 102/500 |
| AT1G52500 | - | 311/500 |
| AT1G52580 | + | 328/500 |
| AT1G52590 | + | 66/500 |
| AT1G53160 | - | 323/500 |
| AT1G54020 | - | 412/500 |
| AT1G55675 | + | 171/500 |
| AT1G56170 | - | 150/500 |
| AT1G58290 | - | 3/500 |
| AT1G63260 | + | 376/500 |
| AT1G64400 | + | 389/500 |
| AT1G64670 | - | 485/500 |
| AT1G66180 | + | 62/500 |
| AT1G66430 | + | 375/500 |
| AT1G67080 | - | 91/500 |
| AT1G68220 | + | 467/500 |
| AT1G68400 | + | 42/500 |
| AT1G68830 | + | 474/500 |
| AT1G69440 | + | 447/500 |
| AT1G69700 | + | 120/500 |
| AT1G69730 | - | 206/500 |
| AT1G70070 | + | 307/500 |
| AT1G70550 | + | 330/500 |
| AT1G71430 | - | 245/500 |
| AT1G71480 | - | 84/500 |
| AT1G71695 | + | 259/500 |
| AT1G72150 | + | 410/500 |
| AT1G72860 | + | 176/500 |
| AT1G74160 | + | 443/500 |
| AT1G74850 | - | 249/500 |
| AT1G75410 | + | 215/500 |
| AT1G75580 | - | 334/500 |
| AT1G75770 | + | 91/500 |
| AT1G75820 | + | 171/500 |
| AT1G76020 | + | 172/500 |
| AT1G76110 | + | 388/500 |
| AT1G78170 | - | 281/500 |
| AT1G78820 | + | 403/500 |
| AT1G80460 | - | 177/500 |
| AT1G80480 | + | 485/500 |
| AT2G01860 | - | 488/500 |
| AT2G02170 | + | 465/500 |
| AT2G02820 | + | 397/500 |
| AT2G02950 | + | 451/500 |
| AT2G03710 | + | 266/500 |
| AT2G04690 | + | 488/500 |
| AT2G05620 | + | 485/500 |
| AT2G12462 | - | 316/500 |
| AT2G13360 | + | 336/500 |
| AT2G16050 | - | 364/500 |
| AT2G16485 | + | 298/500 |
| AT2G17040 | - | 178/500 |
| AT2G17230 | + | 368/500 |
| AT2G17550 | - | 61/500 |
| AT2G18790 | + | 429/500 |
| AT2G19650 | + | 44/500 |
| AT2G19940 | - | 69/500 |
| AT2G22125 | - | 436/500 |
| AT2G22800 | - | 335/500 |
| AT2G25080 | - | 445/500 |
| AT2G25920 | + | 119/500 |
| AT2G26730 | + | 366/500 |
| AT2G27402 | - | 215/500 |
| AT2G28150 | + | 323/500 |
| AT2G28470 | - | 108/500 |
| AT2G29320 | + | 156/500 |
| AT2G29670 | - | 489/500 |
| AT2G31790 | + | 383/500 |
| AT2G32560 | + | 315/500 |
| AT2G33250 | + | 292/500 |
| AT2G33810 | - | 420/500 |
| AT2G34750 | + | 405/500 |
| AT2G35700 | + | 223/500 |
| AT2G35820 | - | 334/500 |
| AT2G38160 | + | 181/500 |
| AT2G38695 | + | 159/500 |
| AT2G38800 | + | 165/500 |
| AT2G39800 | + | 5/500 |
| AT2G41620 | - | 138/500 |
| AT2G41940 | - | 120/500 |
| AT2G42300 | + | 289/500 |
| AT2G42975 | + | 3/500 |
| AT2G43100 | - | 287/500 |
| AT2G43200 | - | 430/500 |
| AT2G43340 | - | 488/500 |
| AT2G43710 | - | 477/500 |
| AT2G44920 | - | 2/500 |
| AT2G45290 | + | 92/500 |
| AT2G45600 | + | 357/500 |
| AT2G46450 | + | 289/500 |
| AT2G46710 | + | 113/500 |
| AT2G46910 | + | 226/500 |
| AT2G46920 | - | 431/500 |
| AT2G47060 | - | 55/500 |
| AT2G47440 | + | 438/500 |
| AT2G47450 | + | 90/500 |
| AT2G47780 | + | 478/500 |
| AT2G47910 | + | 174/500 |
| AT2G47930 | - | 481/500 |
| AT3G01180 | - | 428/500 |
| AT3G01770 | + | 241/500 |
| AT3G02870 | - | 228/500 |
| AT3G04140 | - | 426/500 |
| AT3G04730 | - | 83/500 |
| AT3G05900 | - | 27/500 |
| AT3G06660 | + | 462/500 |
| AT3G06750 | - | 352/500 |
| AT3G09800 | + | 264/500 |
| AT3G10850 | - | 263/500 |
| AT3G11490 | - | 162/500 |
| AT3G11670 | - | 471/500 |
| AT3G11750 | + | 105/500 |
| AT3G15360 | - | 43/500 |
| AT3G15650 | - | 177/500 |
| AT3G15890 | - | 486/500 |
| AT3G16460 | - | 97/500 |
| AT3G17130 | + | 328/500 |
| AT3G20100 | - | 485/500 |
| AT3G20500 | + | 268/500 |
| AT3G21760 | + | 359/500 |
| AT3G21950 | + | 79/500 |
| AT3G23080 | + | 172/500 |
| AT3G23120 | - | 314/500 |
| AT3G23805 | + | 333/500 |
| AT3G25440 | - | 291/500 |
| AT3G26570 | - | 363/500 |
| AT3G26960 | + | 134/500 |
| AT3G30300 | - | 292/500 |
| AT3G47010 | + | 319/500 |
| AT3G51420 | + | 167/500 |
| AT3G51950 | + | 447/500 |
| AT3G52170 | + | 249/500 |
| AT3G52900 | + | 272/500 |
| AT3G57050 | - | 310/500 |
| AT3G58670 | + | 483/500 |
| AT3G58930 | - | 247/500 |
| AT3G59300 | + | 431/500 |
| AT3G61310 | + | 472/500 |
| AT3G61820 | + | 439/500 |
| AT4G00560 | - | 151/500 |
| AT4G01040 | - | 349/500 |
| AT4G01995 | - | 168/500 |
| AT4G03210 | - | 135/500 |
| AT4G04750 | - | 371/500 |
| AT4G04810 | + | 170/500 |
| AT4G08920 | + | 455/500 |
| AT4G09900 | + | 232/500 |
| AT4G09970 | - | 452/500 |
| AT4G10770 | + | 94/500 |
| AT4G15780 | + | 373/500 |
| AT4G15820 | - | 434/500 |
| AT4G16980 | - | 365/500 |
| AT4G17870 | + | 250/500 |
| AT4G19840 | + | 402/500 |
| AT4G20170 | + | 116/500 |
| AT4G21280 | - | 10/500 |
| AT4G22130 | - | 488/500 |
| AT4G22200 | - | 432/500 |
| AT4G22730 | - | 457/500 |
| AT4G23870 | - | 160/500 |
| AT4G24780 | - | 18/500 |
| AT4G25050 | - | 6/500 |
| AT4G25300 | + | 387/500 |
| AT4G25600 | - | 203/500 |
| AT4G31115 | + | 153/500 |
| AT4G31590 | - | 115/500 |
| AT4G31650 | - | 7/500 |
| AT4G32710 | - | 369/500 |
| AT4G34220 | - | 465/500 |
| AT4G34730 | - | 438/500 |
| AT4G35100 | + | 232/500 |
| AT4G36190 | + | 207/500 |
| AT4G36910 | + | 210/500 |
| AT4G37300 | - | 98/500 |
| AT4G37920 | - | 361/500 |
| AT4G38050 | + | 428/500 |
| AT4G38820 | + | 118/500 |
| AT4G38970 | + | 363/500 |
| AT4G39510 | - | 87/500 |
| AT5G01920 | + | 231/500 |
| AT5G02250 | - | 165/500 |
| AT5G03406 | + | 244/500 |
| AT5G04230 | + | 296/500 |
| AT5G05200 | + | 182/500 |
| AT5G06510 | - | 472/500 |
| AT5G06850 | + | 121/500 |
| AT5G07180 | - | 404/500 |
| AT5G07820 | - | 82/500 |
| AT5G09760 | - | 293/500 |
| AT5G10180 | - | 398/500 |
| AT5G10690 | - | 142/500 |
| AT5G12110 | + | 321/500 |
| AT5G13660 | + | 483/500 |
| AT5G13870 | - | 254/500 |
| AT5G14680 | + | 1/500 |
| AT5G16540 | - | 477/500 |
| AT5G16620 | + | 180/500 |
| AT5G17550 | + | 287/500 |
| AT5G17660 | - | 451/500 |
| AT5G18360 | + | 453/500 |
| AT5G19740 | + | 265/500 |
| AT5G21326 | + | 195/500 |
| AT5G21930 | + | 122/500 |
| AT5G22050 | - | 296/500 |
| AT5G22510 | + | 416/500 |
| AT5G23120 | + | 484/500 |
| AT5G23210 | + | 445/500 |
| AT5G23750 | + | 451/500 |
| AT5G23920 | + | 428/500 |
| AT5G25240 | + | 282/500 |
| AT5G26790 | - | 255/500 |
| AT5G28020 | + | 11/500 |
| AT5G33280 | + | 251/500 |
| AT5G37360 | + | 305/500 |
| AT5G38990 | - | 488/500 |
| AT5G39830 | - | 481/500 |
| AT5G40910 | - | 413/500 |
| AT5G42100 | - | 422/500 |
| AT5G42750 | - | 334/500 |
| AT5G42825 | + | 253/500 |
| AT5G43600 | - | 374/500 |
| AT5G43850 | + | 400/500 |
| AT5G44680 | + | 210/500 |
| AT5G45650 | - | 134/500 |
| AT5G45940 | + | 102/500 |
| AT5G46110 | + | 60/500 |
| AT5G46700 | - | 415/500 |
| AT5G47380 | + | 317/500 |
| AT5G47820 | - | 475/500 |
| AT5G48220 | + | 460/500 |
| AT5G48900 | - | 213/500 |
| AT5G51020 | - | 106/500 |
| AT5G51140 | + | 209/500 |
| AT5G51970 | - | 188/500 |
| AT5G53160 | - | 424/500 |
| AT5G53880 | + | 425/500 |
| AT5G53940 | + | 479/500 |
| AT5G55210 | - | 277/500 |
| AT5G55620 | + | 335/500 |
| AT5G57350 | + | 357/500 |
| AT5G58330 | + | 247/500 |
| AT5G58575 | + | 305/500 |
| AT5G59020 | - | 455/500 |
| AT5G59250 | + | 251/500 |
| AT5G60840 | + | 478/500 |
| AT5G62140 | - | 306/500 |
| AT5G63500 | + | 57/500 |
| AT5G63710 | - | 338/500 |
| AT5G65390 | - | 454/500 |
| AT5G66490 | + | 423/500 |
| AT5G67160 | + | 251/500 |
| AT5G67330 | - | 188/500 |
| AT5G67385 | - | 477/500 |

**\*** Overlapping sequences are merged to avoid redundancy. As a result some sequences in the final set may be longer than the specified maximum.

Distribution

+

-

Position values represent number of bases from the 5 prime end of the sequence provided to MEME and the length of that sequence. It is these figures expressed as proportions that are used for statistical analysis.

Probability value of comparison of these values to a linear distribution, p = 0.0025

The motif is found 164 times on the + stand and 130 times on the - strand.

Probability value that it is evenly distributed between the two strands, p = 0.027

Move the mouse pointer over the gene ids to highlight the corresponding marker
